# Comparison of Risk Scores for Predicting Adverse Outcomes in Acute Lower Gastrointestinal Bleeding

**DOI:** 10.1155/2024/3111414

**Published:** 2024-03-21

**Authors:** Chenyang Li, Enqiang Linghu, Chao Chen

**Affiliations:** ^1^Medical School of Chinese PLA, Beijing, China; ^2^Department of Gastroenterology, The First Medical Center of Chinese PLA General Hospital, Beijing, China

## Abstract

**Purpose:**

Acute lower gastrointestinal bleeding (ALGIB) is a common emergency in gastroenterology. Currently, there is insufficient information to predict adverse outcomes in patients with acute lower gastrointestinal bleeding. Our study is aimed at comparing the effectiveness of the clinical risk scores currently utilized and their ability to predict significant outcomes in lower gastrointestinal bleeding.

**Methods:**

We conducted a retrospective observational study of patients who were admitted to ALGIB and underwent colonoscopy or angiography at a single center between January 2018 and December 2022. Adverse outcomes associated with ALGIB included rebleeding, blood transfusion, hemostatic interventions, and in-hospital death. We calculated six risk scores at admission (Oakland, Birmingham, SHA_2_PE, Ramaekers, SALGIB, and CNUH-5). We measured the accuracy of these scores using the area under the receiver operating characteristic curve (AUC) and compared them with DeLong's test.

**Results:**

123 patients with confirmed LGIB (aged 65 years, 55-75) were finally included. The most common diagnoses were colorectal cancer (25%) and hemorrhoids (14%). All scores demonstrated sufficient and comparable effectiveness for hemostatic intervention but no discrimination for rebleeding. The Oakland and SALGIB scores were superior to the other scores in predicting blood transfusion (AUC: 0.97 and 0.95, respectively; *p* = 0.208) and any adverse outcomes (AUC: 0.78 and 0.78, respectively; *p* = 0.854).

**Conclusions:**

The Oakland and SALGIB scores outperform the other scores in predicting the requirement for blood transfusion in ALGIB patients, but no single prediction tool had the best ability across all outcomes. Novel risk stratification scores with higher performance are needed for better risk stratification in ALGIB.

## 1. Introduction

Acute lower gastrointestinal bleeding (ALGIB) is a commonly encountered and possibly fatal disease, accounting for 20-30% of all gastrointestinal bleeding events [1, 2]. In recent years, the incidence of hospitalization due to gastrointestinal bleeding has shown a gradual shift. The incidence of upper gastrointestinal bleeding (UGIB) showed a downward trend, while LGIB experienced a slight increase. The incidence of UGIB in the United States decreased from 112.3 per 100,000 in 2006 to 94.4 per 100,000 in 2014, while the rate of LGIB increased from 146.0 per 100,000 in 2006 to 161.0 per 100,000 in 2015 [3]. Although the majority of patients with acute LGIB can be managed with conservative treatment, about 30% of patients with severe bleeding require inpatient intervention, including blood transfusion, and may receive hemostatic interventions [2].

Several risk scores have been developed to target and prioritize LGIB patients at risk, specifically targeting those who need further intervention due to severe bleeding. However, unlike UGIB, LGIB lacks an available risk score. Although many risk scores for LGIB have been studied, such as BLEED, Strate, HAKA, NOBLADS, Oakland, Birmingham, SHA_2_PE, Ramaekers, SALGIB, and CNUH-5 scores, the heterogeneity in the outcome measures in these studies limits tool comparisons [4–12]. Some scoring systems compromise detailed information, which can be challenging to obtain in the emergency setting.

Insufficient data limit the accuracy of these tools in predicting several adverse consequences, such as recurrent bleeding, the necessity for hemostatic treatment, and blood transfusion. Clinicians require data to precisely target patients at risk due to the heterogeneity of LGIB and the variability of management strategies based on the severity of bleeding. This study is aimed at evaluating and comparing the predictive ability of different clinically validated scores on rebleeding, blood transfusion, hemostatic intervention, and any adverse outcomes in patients with ALGIB.

## 2. Methods

### 2.1. Setting and Participants

A single-center retrospective observational study was conducted in a tertiary center and teaching hospital. This study was approved by the Biomedical Research Ethics Committee of the Fourth Medical Center of Chinese PLA General Hospital, and patient consent was waived due to the retrospective design of the study. Patients aged ≥ 18 years with symptoms suggestive of overt ALGIB (i.e., red or maroon stool, blood mixed with stool, clots per rectum, or passage of melena without hematemesis) who were admitted and underwent colonoscopy or angiography from January 2018 to December 2022 were recruited. Exclusion criteria included age < 18 years, LGIB in patients already hospitalized, confirmed UGIB source, unknown origin, or lack of clinical records including endoscopy or angiography.

### 2.2. Data Collection

Baseline demographics, clinical data (including preexisting medical conditions, vital signs, laboratory results, and prescribed medications), hospital management details, and adverse events (such as rebleeding, blood transfusions, hemostatic interventions, and in-hospital mortality) were extracted from electronic medical records. Endoscopy or angiography was performed depending on individual clinical practice and recorded as the primary procedure. Endoscopy reports were reviewed to determine the source of bleeding. Only definite sources of bleeding were included, defined as lesions with stigmata of recent bleeding (i.e., active bleeding, a visible vessel, or adherent clot), friable tumors, or colitis [2].

### 2.3. Outcomes

The study revealed that the adverse consequences of ALGIB consisted of persistent bleeding within the first 24 h and/or rebleeding, blood transfusion, hemostatic intervention, and in-hospital mortality. Rebleeding was defined as a recurrence of clinically significant bleeding that needed additional blood transfusion, repetition of endoscopic or radiologic procedures, or a further decrease in hematocrit by 20% or more within 24 hours after initial presentation. Blood transfusion was decided according to the recommendations of the National Institute for Health and Care Excellence [13]. The hemostatic intervention was a combination of surgical, endoscopic, and radiologic interventions. In-hospital mortality included deaths attributed to uncontrollable bleeding and severe comorbidities.

### 2.4. Statistical Analysis

The numerical presentation was used for categorical data, whereas quantitative data underwent normality testing via the Kolmogorov–Smirnov test, with nonnormally distributed data presented as the median and interquartile range. Comparisons across groups utilized the Fisher exact test, while continuous data were analyzed through 2-sample *t*-tests. The Oakland, Birmingham, SHA_2_PE, Ramaekers, SALGIB, and CNUH-5 scores were calculated from the data at admission (Online Resource Table 1). Sensitivity, specificity, and Youden's index were calculated to detect the optimal cutoff point. The predictive performance in patients with LGIB was assessed by calculating the AUROC of each scoring system to detect any adverse outcomes [14]. The performance of AUROCs was compared between scoring systems using DeLong's test [15]. *p* < 0.05 was set for statistical significance. All statistical analyses were conducted using version 22.0 of SPSS software (IBM Corporation, Armonk, NY, USA).

## 3. Results

### 3.1. Patient Characteristics and Adverse Outcomes

**Figure 1:**
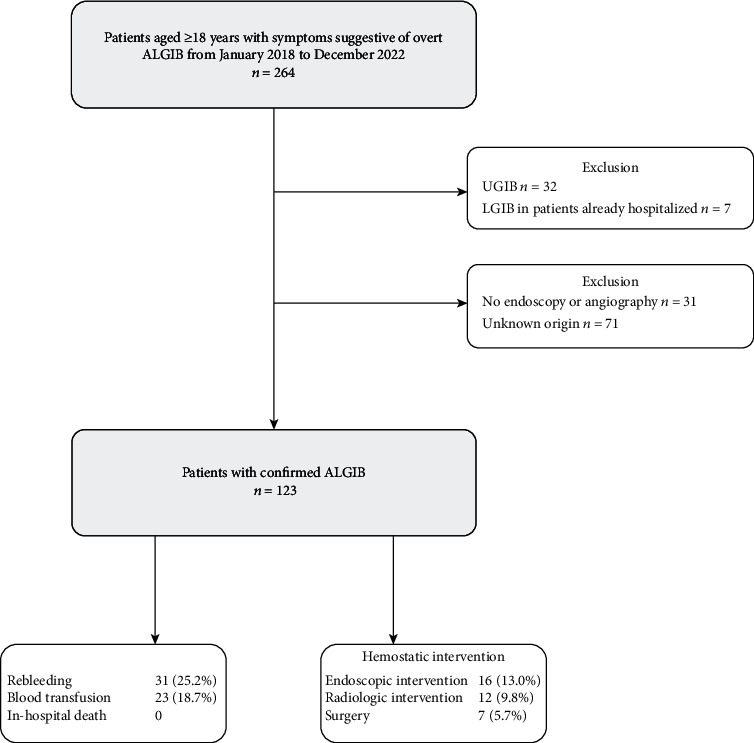
Flowchart of patients recruited for this study. ALGIB: acute lower gastrointestinal bleeding; UGIB: upper gastrointestinal bleeding.

**Figure 2:**
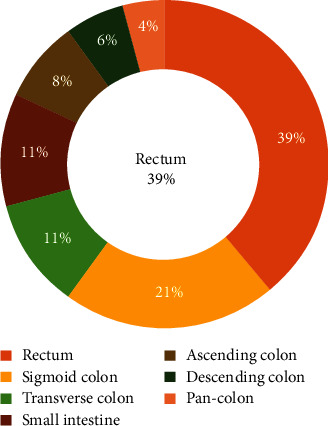
Sites of lower gastrointestinal bleeding.

**Figure 3:**
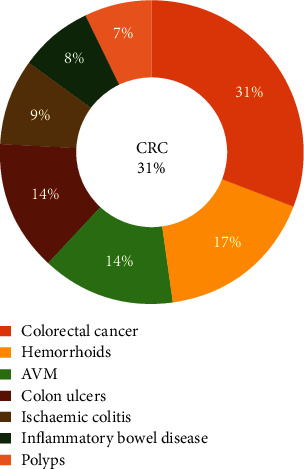
Sources of lower gastrointestinal bleeding. CRC: colorectal cancer; AVM: arteriovenous malformation.

During the study period, ALGIB was diagnosed in a total of 123 patients who underwent colonoscopy or angiography. The cohort had a median age of 65 years, with 60.2% being male.  Table 1 presents the demographic and clinical characteristics of the patients. The most prevalent comorbidities in this cohort were diabetes (56.9%) and hypertension (43.1%). Of the examined patients, 9.8% were taking aspirin, and 5.7% were taking clopidogrel. The adverse events of ALGIB are presented in Figure 1. In our study, we demonstrated the etiology of ALGIB by utilizing Figures 2 and 3. Our findings show that the etiology was detected in 94.3% of patients, with colorectal cancer being the most frequent source (25%), followed by hemorrhoids (14%). Regarding the location of the hemorrhage, 47 (38%) cases exhibited rectal hemorrhage, while 25 (20%) cases exhibited sigmoid hemorrhage. No fatalities occurred during hospitalization, although 47% of patients experienced adverse events. Rebleeding occurred in 25.2% of the cases, and 18.7% needed a blood transfusion. Hemostatic interventions were performed through endoscopic intervention (13%), radiologic intervention (9.8%), and surgery (5.7%).

### 3.2. Comparison of Scoring Systems in Predicting Adverse Outcomes

**Figure 4:**
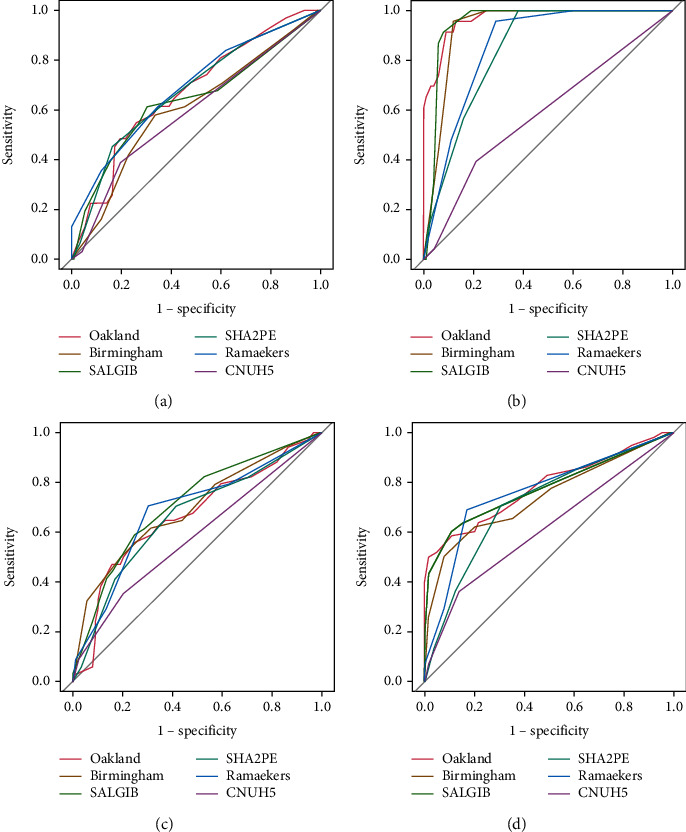
Receiver operating characteristic curves of each score for predicting rebleeding, blood transfusion, hemostatic intervention, and any adverse outcome. (a) Rebleeding. (b) Blood transfusion. (c) Hemostatic intervention. (d) Any adverse outcome.

The Oakland score and SALGIB score both displayed comparable performance in predicting adverse outcomes, yielding an AUC of 0.78 (*p* = 0.854). Conversely, the SHA_2_PE score showed an AUC of 0.72 (*p* = 0.038), and the CNUH-5 score showed an AUC of 0.61 (*p* = 0.001) (Table 2 and Figure 4). Hemostatic intervention delivered acceptable and comparable results across all scores. However, none of the scores were able to sufficiently discriminate rebleeding. The Oakland and SALGIB scoring systems exhibited greater AUC values (0.97 and 0.95, respectively) than the Birmingham (AUC, 0.93), SHA_2_PE (AUC, 0.84), Ramaekers (AUC, 0.86), and CNUH-5 (AUC, 0.59) scores when predicting the need for blood transfusion (all scoring systems, *p* < 0.05). Using Youden's index, cutoff points for predicting undesired results were identified for the Oakland score at 23, SALGIB score at 3, Birmingham score at 6, SHA_2_PE score at 2, and Ramaekers score at 2 (Online Resource Table 2).

## 4. Discussion

Lower gastrointestinal bleeding is a common emergency in gastroenterology departments. Various models have been derived to detect patients at risk of severe bleeding. However, selecting the appropriate tools can be difficult due to the multitude of options available. Conversely, several user-friendly risk scores for UGIB are accessible to guide practice and enhance outcomes [16]. Our study compared multiple risk assessment tools to evaluate their effectiveness in predicting adverse outcomes.

The incidence of adverse effects in our study paralleled that of Tapaskar et al. in the United States [17] but exceeded the rates observed in multiple other cohorts [8, 13, 18, 19]. This discrepancy could be attributed to our inclusion of only verified LGIB cases and the omission of low-risk patients dispatched from the emergency department. No deaths occurred during the study at the hospital, possibly due to the effective treatment of hemostasis provided to most patients with severe bleeding and the exclusion of those with unstable vital signs who were unable to undergo endoscopy or angiography tests. The study underscores the high rebleeding rates after ALGIB, which are significantly higher than those reported in other population-based studies [8, 20]. We think that it was because of the study population's etiology. In our study, colorectal cancer and hemorrhoids were the most common sources of bleeding, which can cause repeated and chronic bleeding. The rate of endoscopic intervention in this study was 13%, which is consistent with the results of other Asian studies [3, 13, 17, 21].

The most prevalent cause of LGIB in this cohort was colorectal cancer, which aligns with the findings of Bai et al. in China [22]. Conversely, it differs from the main cause of diverticular bleeding generally seen in Western nations [23, 24]. Bai et al. analyzed 53951 patients and found no diverticular bleeding. But as same as the Asian population, Quach et al. revealed that diverticular bleeding could be recognized from 6.0% to 8.7% [2, 13], which reminds us that we need further study to figure out the phenomenon. The Oakland score has undergone the most extensive validation for evaluating LGIB. The guidelines in the United States and the United Kingdom for managing LGIB suggest that the Oakland score should be utilized as the leading method for triaging patients [23, 25]. To the best of our knowledge, this study is the initial validation of the Oakland score in the Chinese population. The SALGIB score, recently introduced in Vietnam, can predict severe bleeding in a manner that is comparable to the well-validated Oakland score [13]. Our study found that no single score performed exceptionally well across all outcomes studied. Nevertheless, the Oakland and SALGIB scores were the two best predictors of the need for blood transfusion (AUC: 0.97 and 0.95) and any adverse outcome (AUC: 0.78 and 0.78). The Birmingham score, recently developed in the United Kingdom, incorporates fewer parameters and is thus more user-friendly [9]. Despite being outperformed by the other two scores, our research indicates that the Birmingham score still demonstrates acceptable predictive ability (AUC: 0.74) and blood transfusion (AUC: 0.93). Both the SALGIB and Birmingham scores, due to their fewer components, are more practical for daily use compared to the Oakland score. Other scoring systems are in their early stages of development and consequently lack reliable external validation data. All scores were adequately sufficient and exhibited comparable efficacy for hemostatic treatment, but none displayed discrimination for rebleeding.

The limitations of this study must be acknowledged. It was a retrospective observational study carried out at a solitary center, and its outcomes may differ in hospitals with distinct patient demographics and disease severity. The limited sample size of this study could impede the identification of particular variables or scores that predict adverse events reliably. The study's inclusion criteria solely targeted patients who received colonoscopy, potentially leading to bias. Patients who had been discharged from the emergency department or unable to perform endoscopy or angiography were not taken into consideration, thereby limiting our capability to appraise the efficacy of the tools. Furthermore, multicenter and prospective studies are necessary to compare the effectiveness of risk scores in detecting high- and low-risk patients with ALGIB.

## 5. Conclusions

In conclusion, we compared the ability of several risk scores to predict adverse outcomes in patients with ALGIB. The Oakland and SALGIB scores were superior to the other scores in predicting blood transfusion (AUC: 0.97 and 0.95, respectively) and any adverse outcomes (AUC: 0.78 and 0.78, respectively). The ability of Birmingham and Ramaekers scores to predict any adverse outcomes (AUC: 0.74 and 0.76, respectively) was equivalent to the Oakland and SALGIB scores to some extent. But no single prediction tool had the best ability across all outcomes. Novel risk stratification scores with higher performance are needed for better risk stratification in ALGIB.

## Figures and Tables

**Table 1:** Demographic and clinical characteristics of patients in the study.

Characteristic	Total *n* = 123	No adverse outcome *n* = 65	Any adverse outcome *n* = 58	*p* value
Age, median (IQR)	65 (55, 75)	66 (57, 78)	64 (47, 71)	0.096
Sex				
Male, *n* (%)	74 (60.2)	37 (56.9%)	37 (63.8%)	0.437
Previous admission with LGIB, *n* (%)	40 (32.5)	13 (20.0%)	27 (46.6%)	0.002
Comorbidities				
Heart disease	34 (27.6)	21 (32.3%)	13 (22.4%)	0.221
Stroke	14 (11.4)	6 (9.2%)	8 (13.8%)	0.426
Pulmonary disease	2 (1.6)	1 (1.5%)	1 (1.7%)	>0.999
Liver disease	3 (2.4)	1 (1.5%)	2 (3.4%)	0.601
Renal disease	5 (4.1)	3 (4.6%)	2 (3.4%)	>0.999
Hypertension	53 (43.1)	26 (40.0%)	27 (46.6%)	0.464
Diabetes	70 (56.9)	34 (52.3%)	36(62.1%)	0.443
Cancer	14 (11.4)	6 (9.2%)	8 (13.8%)	0.426
Past medical history of colorectal polyps	8 (6.5)	4 (6.2%)	4 (6.9%)	>0.999
Preadmission medications				0.069
Aspirin	12 (9.8)	10 (15.4%)	2 (3.4%)	
Clopidogrel	7 (5.7)	5 (7.7%)	2 (3.4%)	
Dual antiplatelet	2 (1.6)	1 (1.5%)	1 (1.7%)	
Warfarin	2 (1.6)	1 (1.5%)	1 (1.7%)	
NSAIDs	2 (1.6)	0 (0.0%)	2 (3.4%)	
Corticosteroid	1 (0.8)	1 (1.5%)	0 (0.0%)	
Presenting signs and symptoms				
Clear red bloody stool in the ED	32 (26.0)	8 (12.3%)	24 (41.4%)	<0.001
Blood on DRE	56 (45.5)	29 (44.6%)	27 (46.6%)	0.83
Heart rate (bpm)	77 (72,84)	77 (72, 80)	78 (71, 88)	0.078
SBP (mmHg)	130 (118,142)	133 (120, 148)	127 (114, 137)	0.106
Laboratory data at admission				
White blood cell count (^∗^10⁹/L)	5.93 (4.55,7.20)	5.80 (4.50, 6.90)	6.15 (4.63, 7.28)	0.312
Hemoglobin (g/L)	114 (88,132)	122 (110, 138)	89 (67, 122)	<0.001
Hematocrit (%)	35 (28,40)	37 (33, 41)	28 (22, 36)	<0.001
Platelet (^∗^10⁹/L)	215 (165,262)	212 (165, 260)	215 (164, 261)	0.966
BUN (mmol/L)	5.05 (3.81,6.26)	4.98 (3.93, 5.80)	5.41 (3.77, 6.47)	0.78
Creatinine (*μ*mol/L)	76 (58,89)	77 (62, 90)	72 (57, 86)	0.186
Albumin (g/L)	38.9 (36.0,42.3)	40.3 (37.4, 42.5)	37.9 (33.9, 41.6)	0.017
INR	1.07 (0.99,1.12)	1.03 (0.97, 1.08)	1.11 (1.06, 1.17)	<0.001

Data are *n* (%) or median (IQR: interquartile range). NSAIDs: nonsteroidal anti-inflammatory drugs; ED: emergency department; DRE: digital rectal examination; LGIB: lower gastrointestinal bleeding; SBP: systolic blood pressure; BUN: blood urea nitrogen.

**Table 2:** Performance of different risk scores in comparison with the Oakland score in the prediction of adverse outcomes.

	Rebleeding	Blood transfusion	Hemostatic intervention	Any adverse outcome
	*n* = 31 (25.2%)	*n* = 23 (18.7%)	*n* = 35 (28.5%)	*n* = 58 (47.2%)
Oakland	0.67 (0.56-0.78)	0.97 (0.94-0.99)	0.66 (0.55-0.78)	0.78 (0.70-0.87)
Birmingham	0.60 (0.48-0.72)	0.93 (0.89-0.97)	0.70 (0.59-0.80)	0.74 (0.65-0.83)
	*p* = 0.004	*p* = 0.019	*p* = 0.183	*p* = 0.053
SALGIB	0.64 (0.52-0.76)	0.95 (0.91-0.99)	0.71 (0.61-0.81)	0.78 (0.70-0.86)
	*p* = 0.331	*p* = 0.208	*p* = 0.144	*p* = 0.854
SHA_2_PE	0.68 (0.57-0.78)	0.84 (0.78-0.91)	0.66 (0.55-0.77)	0.72 (0.63-0.81)
	*p* = 0.891	*p* < 0.001	*p* = 0.922	*p* = 0.038
Ramaekers	0.69 (0.58-0.80)	0.86 (0.80-0.92)	0.69 (0.58-0.79)	0.76 (0.68-0.85)
	*p* = 0.698	*p* < 0.001	*p* = 0.546	*p* = 0.518
CNUH-5	0.59 (0.50-0.69)	0.59 (0.48-0.70)	0.58 (0.49-0.67)	0.61 (0.54-0.69)
	*p* = 0.185	*p* < 0.001	*p* = 0.24	*p* = 0.001

Data are presented as areas under the receiver operating characteristic curve and 95% confidence intervals; *p* values are from the DeLong et al. test.

## Data Availability

All data generated or analyzed during this study are included in this article. Further enquiries can be directed to the corresponding authors.

## Supplementary Materials

Supplementary 1 Online Resource Table 1: components of the Oakland, SALGIB, Birmingham, SHA_2_PE, Ramaekers, and CNUH-5 scores.

Supplementary 2 Online Resource Table 2: cutoff points of different scores for predicting the presence of any adverse outcome.
