# Supplementary figures and images for: Comparison of Risk Scores for Predicting Adverse Outcomes in Acute Lower Gastrointestinal Bleeding
Source: Gastroenterol Res Pract. 2024 Mar 21;2024:3111414. doi: 10.1155/2024/3111414 (PMC10978071; doi:10.1155/2024/3111414)

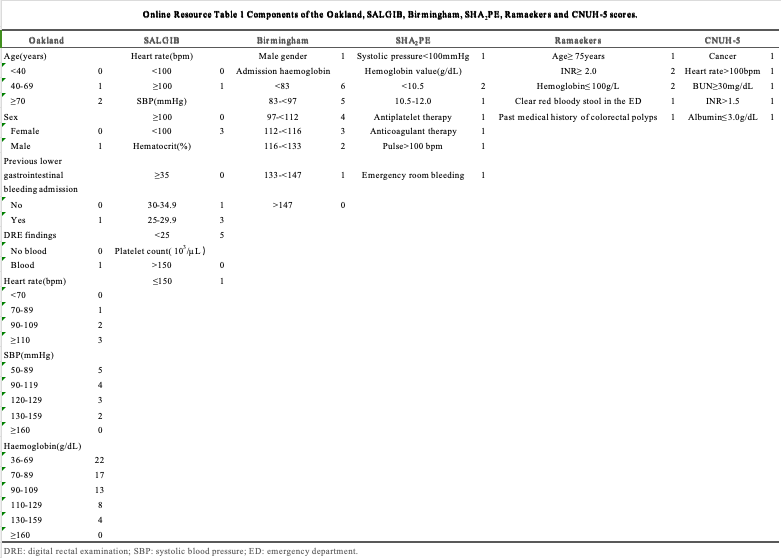

Supplement: Supplementary 1 — Online Resource Table 1: components of the Oakland, SALGIB, Birmingham, SHA2PE, Ramaekers, and CNUH-5 scores. [file 3111414.f1.png]

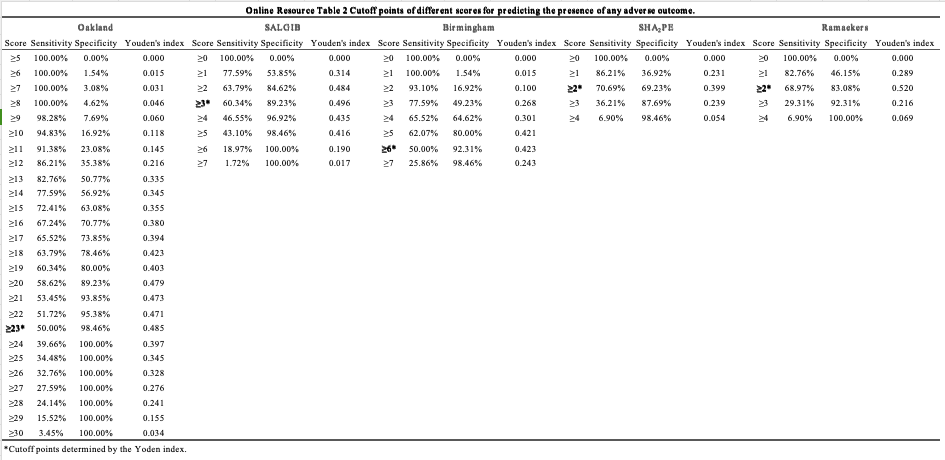

Supplement: Supplementary 2 — Online Resource Table 2: cutoff points of different scores for predicting the presence of any adverse outcome. [file 3111414.f2.png]
